# Development of a computer-based quantification method for immunohistochemically-stained tissues and its application to study mast cells in equine wound healing (proof of concept)

**DOI:** 10.1186/s12917-020-02444-x

**Published:** 2020-07-02

**Authors:** Valérie Dubuc, Sheila Laverty, Hélène Richard, Monique Doré, Christine Theoret

**Affiliations:** 1 Department of Veterinary Biomedicine, Faculté de Médecine Vétérinaire, Université de Montréal, 3 200 Sicotte, Saint-Hyacinthe, Québec, Canada; 2 Department of Clinical Sciences, Faculté de Médecine Vétérinaire, Université de Montréal, 1 500 des Vétérinaires, Saint-Hyacinthe, Québec, Canada; 3 Department of Pathology and Microbiology, Faculté de Médecine Vétérinaire, Université de Montréal, 3 200 Sicotte, Saint-Hyacinthe, Québec, Canada

**Keywords:** Immunohistochemistry, Quantification, ImageJ, Horse, Skin, Wound healing, Mast cell

## Abstract

**Background:**

There is a growing interest in the scientific community to use computer-based software programs for the quantification of cells during physiological and pathophysiological processes. Drawbacks of computer-based methods currently used to quantify immunohistochemical staining are the complexity of use, expense of software and overly-simplified descriptions of protocol thereby limiting reproducibility. The precise role of mast cells in equine cutaneous wound healing is unknown. Given the contribution of mast cells to the chronic inflammation observed in human keloid, a pathology similar to exuberant granulation tissue (EGT) in horses, mast cells might be present in high numbers in equine limb wounds predisposed to EGT. The main goal of this study was to develop a reliable and reproducible quantification method for immunostained tissues using a computer software that is widely available, at no cost, to the scientific community. A secondary goal was to conduct a proof of concept using the newly-established method to quantify mast cells during wound healing at different anatomical sites (body and limb) in horses to see if a different pattern is observed in limb wounds, which are predisposed to EGT.

**Results:**

A good intraclass correlation coefficient (ICC, 0.67 *p* < 0.05) was found between the computer-based ImageJ method and manual counting. An excellent intra-operator ICC of 0.90 (*p* < 0.01) was found for the ImageJ quantification method while a good interoperator ICC of 0.69 (*p* < 0.01) was measured. No significant difference was observed between the variation of the ImageJ and that of the manual counting method. Mast cells were localized below the epidermis, around cutaneous appendages and blood vessels. Mast cell numbers did not differ significantly in relation to anatomical location or time of healing.

**Conclusions:**

The computer-based quantification method developed is reliable, reproducible, available, cost-free and could be used to study different physiological and pathological processes using immunohistochemistry.

## Background

Major drawbacks of computer-based methods currently used to evaluate immunohistochemically-stained tissues are the complexity of use, expense of software and overly-simplified descriptions of protocol limiting reproducibility [1]. Another challenge is to ensure objectivity by validating the method with appropriate parameters including reliability, repeatability and reproducibility [2]. The development of new computer-based methods would be useful to facilitate quantification of cellular populations in immunostained tissues.

Wounds on the limbs of horses suffer a weak and prolonged inflammatory response compared to body wounds, that impairs healing and may lead to the development of exuberant granulation tissue (EGT) [3]. Mast cells are suspected to contribute to chronic inflammation, as is the case in human keloid, a fibroproliferative pathology exhibiting features comparable to EGT [4].

Mast cells are immune cells that secrete vasodilating factors, and numerous pro- and anti-inflammatory cytokines during wound healing [5]. They also express a c-kit receptor (CD117) that enables cell communication with fibroblasts. Their precise role in cutaneous wound healing in horses is unknown.

The objectives of this study were to 1) develop a reliable and reproducible quantification method for immunostained tissues using a computer software that is widely available, at no cost, to the scientific community, and 2) conduct a proof of concept using the newly-established method to quantify mast cells during skin wound healing at different sites (body and limb) in horses in an effort to determine if a different mast cell pattern is observed in limb wounds predisposed to EGT. It was hypothesized that 1) the computer-based method developed would have excellent repeatability, reproducibility and reliability parameters, and 2) mast cell numbers would be greater and would persist in limb wounds compared to body wounds throughout healing, since this cell is known to contribute to chronic inflammation in human keloids.

## Results

**Fig. 1:**
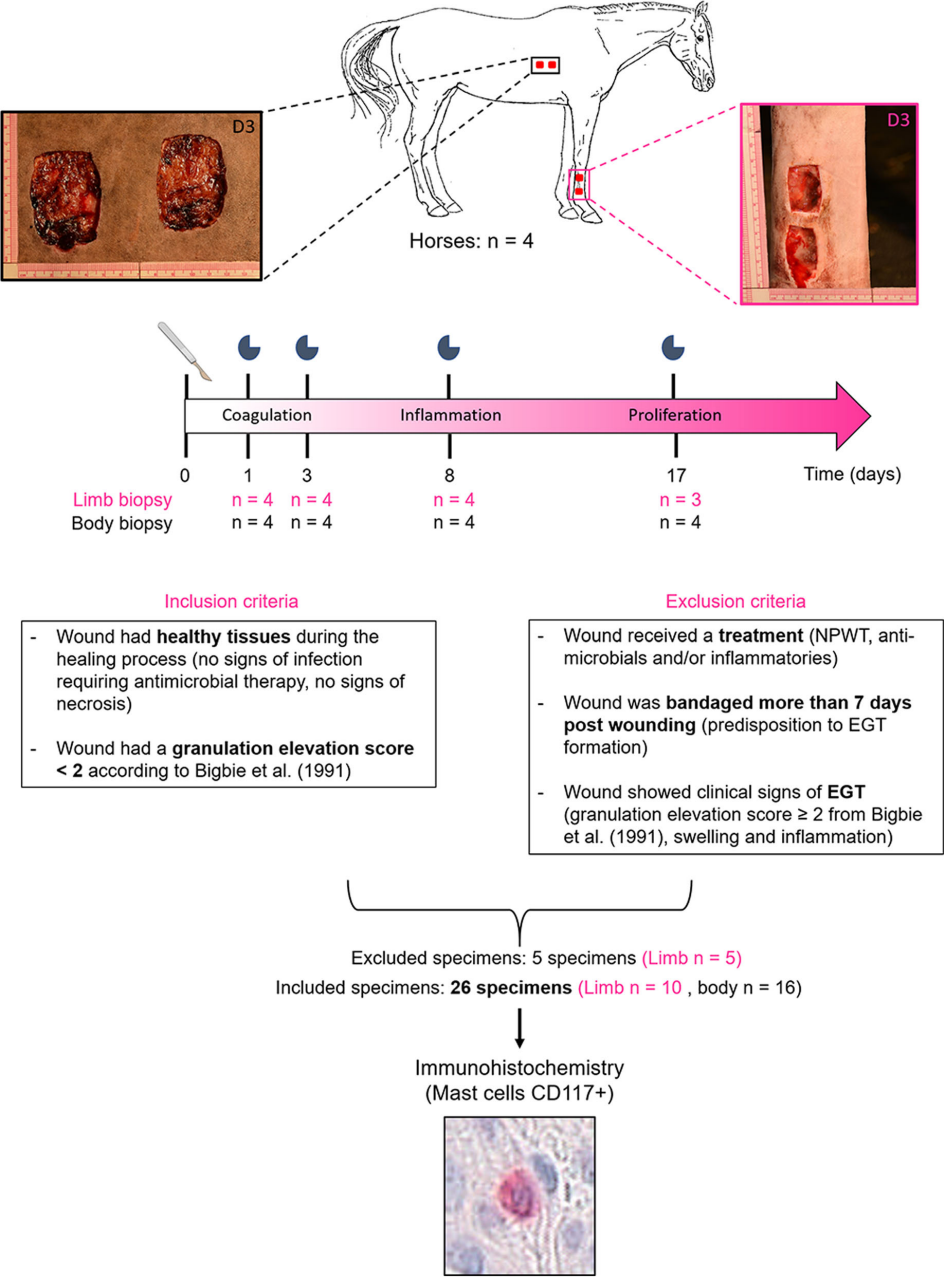
Study design. Full-thickness excisional wounds were created on the thorax (15 cm^2^) and the forelimb (6 cm^2^) (2 per anatomical site) on 4 mixed breed mares and left to heal by second intention. Samples were harvested alternatively between the two wounds/site on days 1, 3, 8 and 17. Based on inclusion and exclusion criteria, a total of 26 healthy wounds were included. Two specimens showed clinical signs of EGT and 3 others received NPWT treatment, all harvested from limb wounds, and were thus excluded. The photomicrograph was taken at a 400x magnification. NPWT: negative pressure wound therapy, EGT: exuberant granulation tissue

One mare developed EGT on its limb wounds from 11 days post wounding until the end of the study and these specimens were excluded from the final analysis (Fig. 1, Additional file 4: Table S1). A total of 16 body and 10 limb wound specimens were included for a total of 26 normally healing wound specimens (Fig. 1).

### Validation of the ImageJ computer-based method

**Fig. 2:**
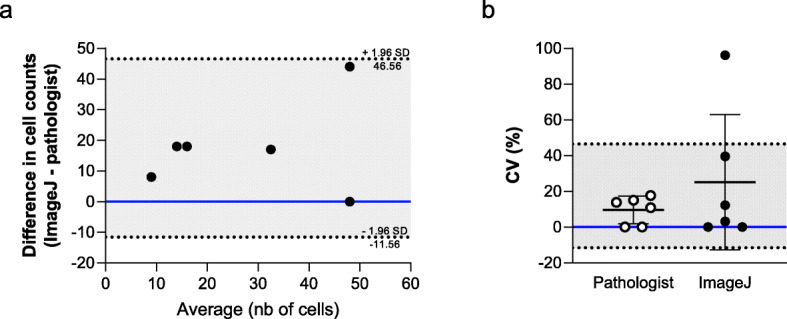
Comparison of the variation between the ImageJ and the pathologist manual counting method. **a** Bland-Altman plot of the difference in cell counts between the ImageJ method and the pathologist manual counting method compared to the average (*n* = 6). The difference in cell counts corresponds to the difference between the mean of two counts done by each method. **b** Data represent the coefficient of variation expressed in percentage between two counts. White circles represent the pathologist manual-based counting method and black circles represent the ImageJ computer-based method. Statistical analysis was done on paired data for each specimen (*n* = 6). Lines and bars represent the mean ± standard deviation (SD). Upper and lower dashed lines correspond to the upper and lower 95% agreement limits. %CV: coefficient of variation in percentage

The validation of the ImageJ computer-based method was performed on 6 specimens. An intraclass correlation coefficient (ICC) value of 0.67 (*p* < 0.05) was found between the ImageJ method and the pathologist-based manual counting (Table 1). An intra-operator ICC of 0.90 (*p* < 0.01) and an inter-operator ICC of 0.69 (*p* < 0.01) were found for the computer-based method (Table 1). The raw data indicate, that a slight overestimation of mast cell counts occurred with the ImageJ method compared to the manual counting method and may relate to the exclusion of cells, by the pathologist, based on observable morphological parameters (Fig. 2a). Coefficients of variation were calculated for the ImageJ computer-based and the pathologist manual counting-based methods to compare the variation between two counts of each method (Fig. 2b). A greater variation was observed for the ImageJ method (25.19 ± 37.86%) compared to the manual counting method (9.58 ± 7.73%), but no statistically significant difference was observed (*p* = 0.69).

**Table 1:** Validation of the quantification method using ICCs (*n* = 6)

Comparisons	ICC	p
Same operator	0.90	0.00
Two different operators	0.69	0.01
Mean of ImageJ vs pathologist	0.67	0.04

Pathologist: manual counting; *ICC* Intraclass correlation coefficient; The ICC calculated between the ImageJ method and the pathologist-based manual counting method used the mean of the 2 counts done by each method *p* < 0.05 was considered statistically significant

### Proof of concept: quantification of mast cells

**Fig. 3:**
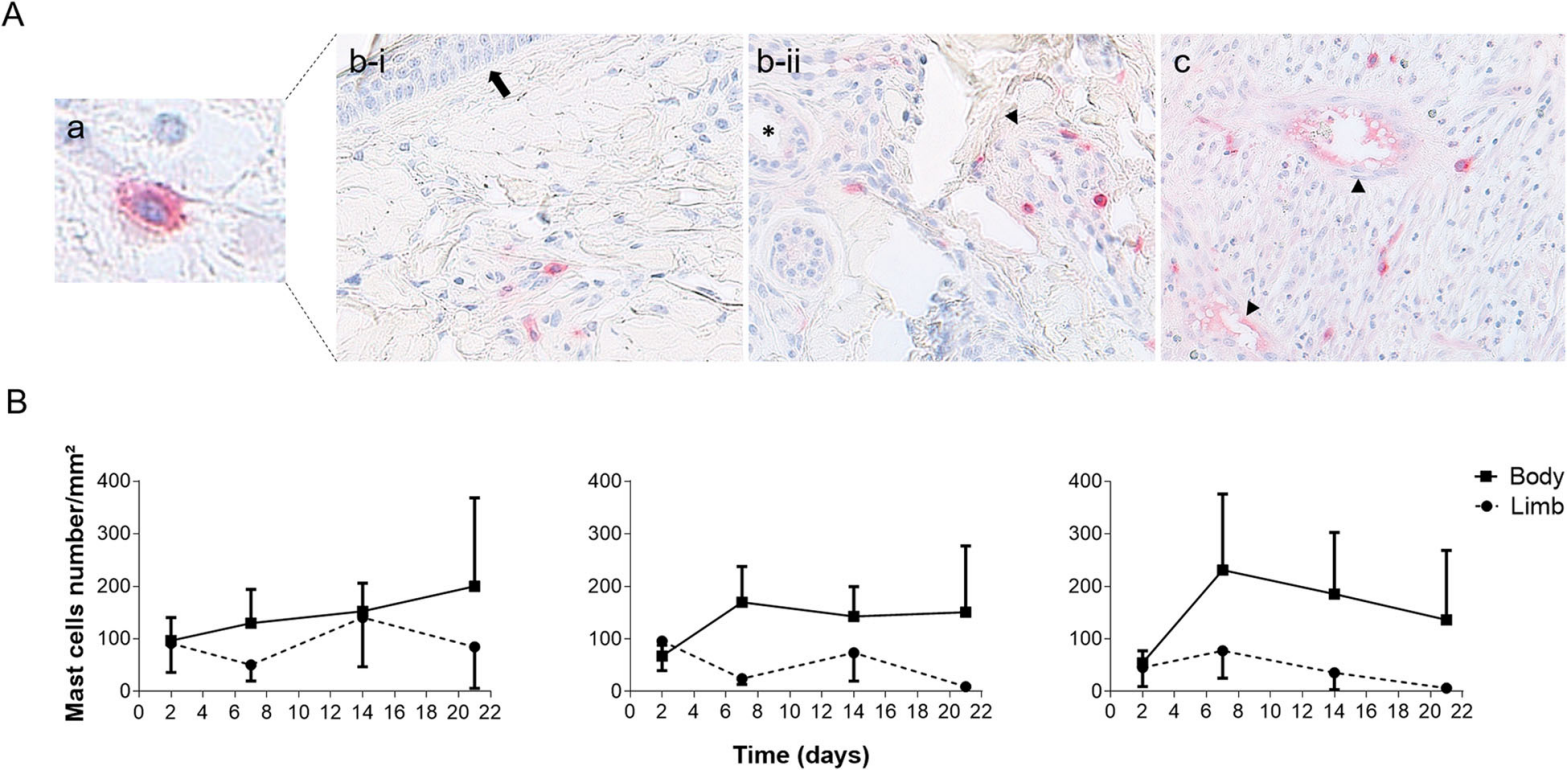
Photographs and quantification of CD117+ mast cells in skin wounds of horses. **a** Photographs showing a a) HPF of an equine CD117+ skin mast cell (400x) and b) mast cells distribution in the i-subepidermal and ii-deep dermal layer of the intact skin borders and in the c) granulation tissue of a 3-day old thoracic wound. Immunohistochemistry was performed with the avidin-biotin-alkaline phosphatase method and used Harris hematoxylin counter stain (× 200). Arrow: epidermis, arrowhead: blood vessel, asterisk: sweat gland. **b** Graphs showing evolution of mast cells numbers (/mm^2^) over time and according to anatomical location in the subepidermal layer, deep dermal layer, and granulation tissue (left to right). Values represent mean ± standard error of the mean (SEM) (*n* = 26)

Mast cells in intact skin borders were located in the subepidermal and deeper dermal layers and were mostly found around hair follicles, sweat and sebaceous glands, and blood vessels (Fig. 3A-b). Mast cells were also well distributed throughout the granulation tissue (Fig. 3A-c).

In the subepidermal and deep dermal layer, mast cell numbers were greater in body wounds and more variability was seen in limb wounds, but these changes were not statistically significant (Fig. 3B). In the granulation tissue, mast cell kinetics between body and limb wounds were similar, except that the magnitude of the reaction seemed inferior in limb wounds (not statistically significant) (Fig. 3B).

## Discussion

The primary objective of this study was to develop a reliable and reproducible quantification method based on a widely-available cost-free computer software, for the objective evaluation of immunohistochemically-stained tissues. The ImageJ computer-based method developed in this study allows numerical analysis of cells with cell numbers (number of cells/mm^2^ of tissue) as opposed to previously developed computer-based quantification methods allowing the quantification of the immunostaining intensity [6].

The intra-operator ICC value confirmed excellent repeatability of the ImageJ method while inter-operator ICC value showed good reproducibility [7], suggesting that reliability is improved if the method is used by a single operator. This is likely due to the subjective nature of the manual subtraction step required to delete structures considered to be stained non-specifically (Fig. 5d). Few histomorphology studies have validated newly-developed computer-based quantification methods with ICCs to compare the reliability of the methods with the evaluation done by a pathologist or a tissue specialist (e.g. surgeon), mostly considered as the gold standard method [8–12]. To the authors’ knowledge, this study is the first to develop and validate a computer-based quantification method, with ICCs, to evaluate equine wound healing. The ICC found between the ImageJ and the pathologist-based methods (0.67; *p* < 0.05) confirmed good reliability between the ImageJ and the gold standard methods [13]. Results from this study show no difference in the coefficients of variation between the two methods. The greater mean of coefficients of variation observed in the ImageJ method group could be explained by a single specimen having a coefficient of variation of 96% between count 1 and 2, thus significantly increasing the mean of the group. Other data had a coefficient of correlation between 0 and 40% and had a minimal and maximal difference of 3 and 30%, respectively, with the pathologist group. The manual subtraction step can bring subjectivity and therefore variation between one count to another for the ImageJ method, but the excellent intra-operator ICC found shows that measurements are repeatable. It is difficult to compare such results with other studies aiming to develop computer-based methods applied to cutaneous wound healing since these studies are scarce and ICC agreement intervals may differ in the literature [7, 9, 13, 14]. In short, the hypothesis is partially confirmed since an excellent repeatability and good reproducibility and reliability were found. In light of these results, the newly-developed computer-based ImageJ method presents advantages over manual counting. It yields a precise number of cells in a defined area of tissue and a cell can not be counted twice, limiting human error. Importantly, it requires less expertise and can be used by a variety of personnel when a pathologist or a wound specialist is not available, and it has fewer subjective steps in its process. The ImageJ computer-based method has the manual subtraction step that can introduces subjectivity, while this step as well as the inclusion or exclusion of mast cells contribute to subjectivity in the manual-based counting method. The inclusion or exclusion of mast cells in the counting for the ImageJ method is based on a mathematical parameter (cell area) that is less subjective than morphological parameters like cell shape, visual cell size and nucleus/cytoplasm ratio. It is recognized that in tissues with small numbers of immunostained cells, manual counting could be satisfactory. The advantages discussed above could also make this method a suitable tool for the quantification of other immunostained inflammatory cells like neutrophils and macrophages during physiological and pathological processes. With specific antibodies, the software program can analyze tissues with either numerous or few stained cells since it recognizes the IHC staining. Other mathematical parameters can also be added to the method like the circularity parameter to allow the quantification of multilobed nuclei of cells such as neutrophils [15, 16]. The ImageJ computer-based method we described could eventually help toward monitoring wound healing and response to therapy in the future by the measurement of the immunostained cells present in a small wound biopsy to enhance care.

As a proof of concept, the newly-developed ImageJ method was used to quantitate mast cells, for the first time, in specimens harvested from healing skin wounds of horses. The location of mast cells in the subepidermal and deep dermal layers agrees with other studies conducted on equine skin (Fig. 3A-b) [17, 18]. However, the scattered distribution within the granulation tissue of equine wounds has, to our knowledge, never been reported (Fig. 3A-c). The smaller numbers observed in limb compared to body wounds (Fig. 3b) may reflect a weak inflammatory response seen in limb wounds of horses, refuting our hypothesis [3]. Additional studies are needed to characterize the intrinsic properties of equine mast cells by cell culture and/or other laboratory techniques such as flow cytometry and ELISA to better understand their role in cutaneous wound healing. Although the role of mast cells have been investigated in porcine models where a hypercontractive model was treated with ketotifen (a mast cell stabilizer) and showed significantly less wound contraction than its nontreated control, much remains unknown [19, 20]. Eventually, it may be interesting to compare the kinetics and the number of mast cells of naturally healing equine wounds with those affected by EGT in an effort to establish reference values, a cut off value of mast cell number indicating poor wound prognostic and the clinical role of this cell type.

The principal limitation of our study is the small number of horses, which may explain why no significant differences were found between limb and body wounds in the proof of concept portion of the study, since mast cell numbers are known to show great interindividual variation in horses [17]. However, the multiple sites and the longitudinal nature of the study added robustness for statistical analysis. Results obtained here should help to elucidate the role of mast cells in normal wound healing and future studies might include EGT-affected wounds to document the numbers of mast cells in this healing impairment common to horses.

## Conclusions

In conclusion, a reliable, reproducible, available and cost-free computer-based method has been developed to objectively evaluate immunostained tissues. This method could be useful to study and quantify several types of cells implicated in physiological and pathophysiological processes.

## Methods

### Samples

Specimens were sourced from a wound tissue bank repository, harvested and archived from four mixed breed mares included in a previous controlled and randomized study [21]. The study’s protocol was approved IACUC of the Université de Montréal and followed the guidelines of the Canadian Council on Animal Care (approval # 15-Rech-1811). Briefly, four full-thickness skin wounds were created, after sedation and local anesthesia, on one lateral mid-thoracic wall (two 15 cm^2^ wounds) and on the distal extremity of one thoracic limb (two 6 cm^2^ wounds) per horse (corresponding to the control wounds in the original study). Wounds were left to heal by second intention. Full-thickness wound samples were harvested with an 8 mm diameter biopsy punch from the edge of the 2 wounds on the limb and on the thoracic wall (alternatively) at day 1 (D1), D3, D8 and D17. D0 samples corresponded to normal skin obtained upon wound creation. Wounds and granulation tissue were photographed during the healing process and photographs were visually evaluated at the end of the study for granulation tissue elevation scoring according to the Bigbie et al. scale by a blinded board-certified veterinary surgeon. Elevation scores between 1 and 4 were attributed, score 1 corresponded to an elevation not exceeding the edges of the wound. Score 2 to an elevation at the same level as the wound edge. Score 3 corresponded to an elevation exceeding the edges of the wound. Score 4 corresponded to an elevation exceeding the edges of the wound and covering the epithelium [22]. The experimental model is shown in Fig. 1. A total of 31 specimens were harvested and archived, of which 26 were included in the current study based on inclusion and exclusion criteria (*n* = 26) (Fig. 1, Additional file 4: Table S1) [22]. Specimens included 3–4 mm of intact skin borders (subepidermal layer and the deep dermal layer) and 3–4 mm of the wound (granulation tissue) and were fixed in 10% formalin, dehydrated, paraffin-embedded and then cut into 4 μm sections [21].

### Immunohistochemistry for mast cell staining

**Fig. 4:**
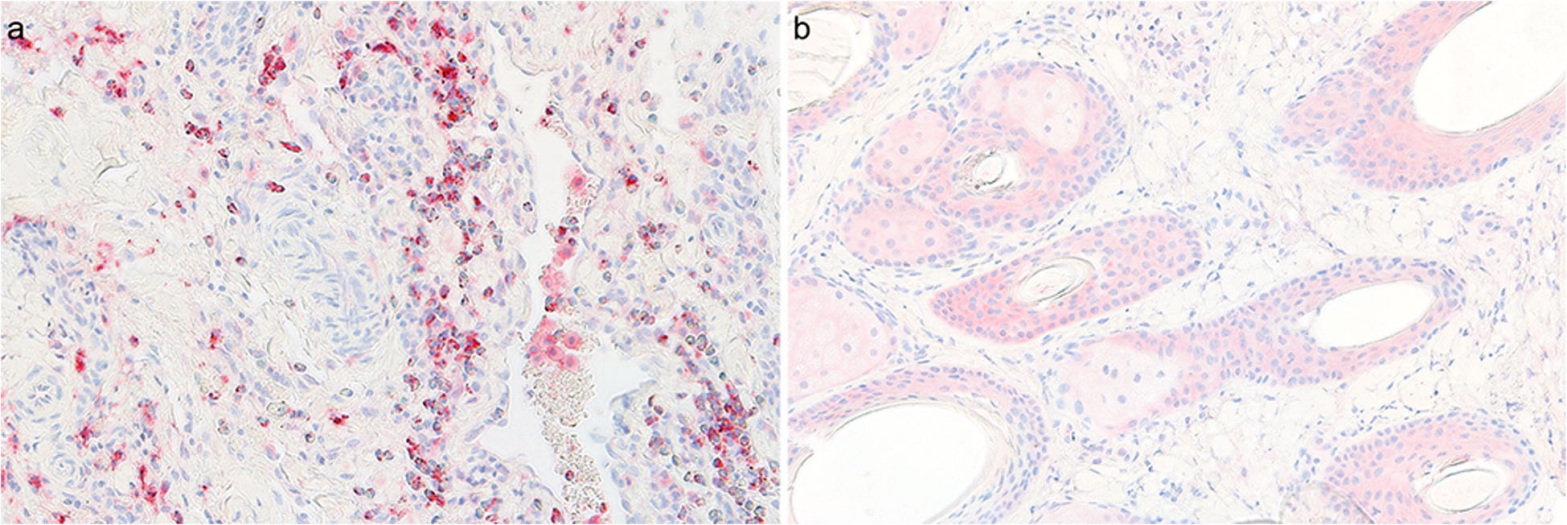
Positive and negative controls included in the immunohistochemistry technique. **a** Positive control showing mast cells in an equine mast cell tumor. **b** Negative control using rabbit serum for the replacement of the primary antibody on a 3-day old limb wound. No cellular staining was observed but unspecific staining of epidermis, sebaceous and sweat glands, and hair follicles, was noted. An additional blocking step of the endogenous biotin was added to the protocol and limited the unspecific staining. The technique used the avidin-biotin-alkaline phosphatase method with Harris hematoxylin counter stain (× 200)

A rabbit polyclonal anti-CD117/c-kit antibody (1/87, #RB-9038, Thermo Fisher Scientific, Rockford, IL, USA) was incubated on the sections for 1 h at room temperature [23]. An equine mast cell tumor was used as a positive control and rabbit serum replaced the primary antibody for the negative control (Fig. 4). The detailed protocol is presented in Additional file 1.

### Image analysis and quantification

**Fig. 5:**
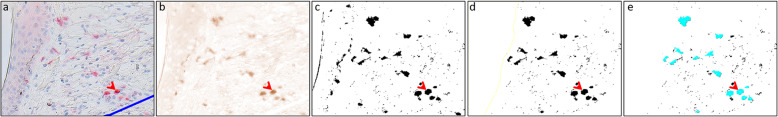
Principal steps of mast cell quantification protocol using ImageJ software program. **a** Original photo illustrating the subepidermal layer of a one-day old skin wound on the limb stained with the CD117 antibody. The blue line represents the border between the subepidermal and the deeper dermal layer. **b** Staining of CD117+ mast cells after colour deconvolution and **c** its binary transformation. **d** Manual subtraction of stained cutaneous appendages; the deeper dermal region below the blue line was also deleted. **e** Remaining cells stained by the antibody; blue cells are those included in the count by the software (cell area between 8 and 300 μm^2^). The original photo was taken at a 200X magnification. Arrow head points to a stained mast cell

Immunostained cells were observed under light microscopy at 200x and scanned with the Panoptiq software program v.1. 4. 3 (ViewsIQ, Richmond, BC, CAN). Mast cell characterization and quantification was performed by 2 blinded observers in the wound samples harvested from a former study [21]. In the intact skin borders of the wound, mast cell quantification was performed in the subepidermal layer (0–296 μm under basal membrane) and in the deep dermal layer (296–1184 μm) [18]. In the wound, mast cell quantification was performed in the granulation tissue. From each region, 5 high power fields (HPFs) were selected randomly. Mast cells were quantified using the newly-developed computer-based quantification method based on the ImageJ software program (U. S. National Institutes of Health, Bethesda, MD, USA). Mast cells in six of these specimens were also manually counted by an ACVP-certified pathologist and statistically compared to the ImageJ method. For both methods, identification of mast cells was based on CD117+ staining by immunohistochemistry and the presence of a nucleus. Particles non-specifically-stained and small foci of non-specific staining without a nucleus were manually (ImageJ) or visually (pathologist) subtracted and excluded from the count. For the ImageJ method, CD117+ cells with a cell area between 8 to 300 μm^2^ were considered mast cells and were included in the count. For the pathologist-based manual counts, inclusion of mast cells in the counts was based on the following morphological parameters: cell shape, cell size and nucleus/cytoplasm ratio. The computer-based quantification method is shown in Fig. 5 and the complete protocol is described in Additional file 2. Additional file 3 provides details about the chosen area range. Mast cell numbers were then compared between limb and body wounds.

### Statistical analysis

Statistical analysis was done with SPSS software program v.25 (IBM, Armonk, NY, USA) to validate the quantification method by calculating ICCs and Wilcoxon signed rank test for paired data, and with SAS software program v.9.3 (SAS Institute Inc., Cary, NC, USA) for mast cell quantification. A linear model for repeated measures was used to detect differences relating to time and to anatomical location of the wound (limb vs body). A *p*-value < 0.05 was considered statistically significant.

## Supplementary information

**Additional file 1.** Detailed mast cell immunohistochemistry protocol. Full immunohistochemistry protocol for the mast cell identification in limb and body wounds of horses included in the study.

**Additional file 2.** ImageJ quantification protocol used on mast cells. Full protocol of the quantification of mast cells in limb and body wounds of horses included in the study with the ImageJ software program.

**Additional file 3.** Establishment of protocol of mast cell area interval. This document illustrates how the range of mast cell area interval was established by using the ImageJ software program for the later quantification.

**Additional file 4 Table S1.** Numbers and characteristics of the samples included in the study. Specimen characteristics are grouped in a table to show which specimens were included or excluded in the study and why.

## Data Availability

The datasets used and/or analyzed during the current study are available from the corresponding author on reasonable request.

## Abbreviations

EGT: Exuberant granulation tissue
ICC: Intraclass correlation coefficient
NPWT: Negative pressure wound therapy
IHC: Immunohistochemistry
HPF: High power field
