# Supplementary material for: Development of a computer-based quantification method for immunohistochemically-stained tissues and its application to study mast cells in equine wound healing (proof of concept)
Source: BMC Vet Res. 2020 Jul 2;16:228. doi: 10.1186/s12917-020-02444-x (PMC7330934; doi:10.1186/s12917-020-02444-x)
Supplement: Supplementary file 1 — Additional file 1. Detailed mast cell immunohistochemistry protocol. Full immunohistochemistry protocol for the mast cell identification in limb and body wounds of horses included in the study. [file 12917_2020_2444_MOESM1_ESM.docx]

**Supplementary information 1**

***Detailed mast cell immunohistochemistry protocol***

Specimens were dewaxed in xylene (4 x 5 minutes) and rehydrated in alcohol grades from 100% to 70% (2 minutes each). After a 5 minute wash in distilled water, sections were heated in a 1 mM sodium citrate buffer solution at pH = 6.0 (#S464, Sigma-Aldrich, Saint Louis, MO, USA) at 95°C for 30 minutes for antigen unmasking. This step was followed by a cooling period of 15 minutes at room temperature, 3 rinses in distilled water and 3 washes in PBS (3 x 2,5 minutes). Sections were then incubated with 10% goat serum (#053110, Multicell Wisent, St-Bruno, QC, CAN) prepared in PBS containing 1% w/v BSA for 30 minutes at room temperature to avoid unspecific binding of the secondary antibody. After a brief wash in PBS, an endogenous biotin blocking solution (#X0590, Dako, Mississauga, ON, USA) was incubated on the sections as per manufacturer’s instructions. A brief wash in PBS was done and a rabbit polyclonal anti-CD117/c-kit (#RB-9038, Thermo Fisher Scientific, Rockford, IL, USA) prepared 1/87 in PBS and 1% w/v BSA was incubated on the sections for 1 hour at room temperature. Rabbit serum replaced the primary antibody for the negative control. After 3 washes in PBS, an IgG goat anti-rabbit secondary antibody conjugated with biotin (#31822, Vector Laboratories, Burlingame, CA, USA) prepared in PBS and 1% w/v BSA was incubated 1/300 on the sections for 45 minutes at room temperature. This was followed by 3 washes in PBS and a 45 minute incubation at room temperature of an alkaline phosphatase solution from a Vectastain® ABC-AP staining kit (AK-5000, Vector Laboratories, Burlingame, CA, USA) prepared according to the manufacturer’s instructions. After 3 washes in PBS, sections were incubated with Vector Red (SK-5100, Vector Laboratories, Burlingame, CA, USA) for a period of 20 minutes in the dark and prepared according to the manufacturer instructions. The nuclei were stained with Harris hematoxylin and, finally, slides were mounted with Micromount (Leica Biosystems, Richmond, IL, USA). An equine mast cell tumour was used as a positive control.
