# Supplementary material for: Development of a computer-based quantification method for immunohistochemically-stained tissues and its application to study mast cells in equine wound healing (proof of concept)
Source: BMC Vet Res. 2020 Jul 2;16:228. doi: 10.1186/s12917-020-02444-x (PMC7330934; doi:10.1186/s12917-020-02444-x)
Supplement: Supplementary file 2 — Additional file 2. ImageJ quantification protocol used on mast cells. Full protocol of the quantification of mast cells in limb and body wounds of horses included in the study with the ImageJ software program. [file 12917_2020_2444_MOESM2_ESM.docx]

**Supplementary information 2**

***Mast cells ImageJ quantification protocol***

**1.0 Mast cell counting**

- 1.
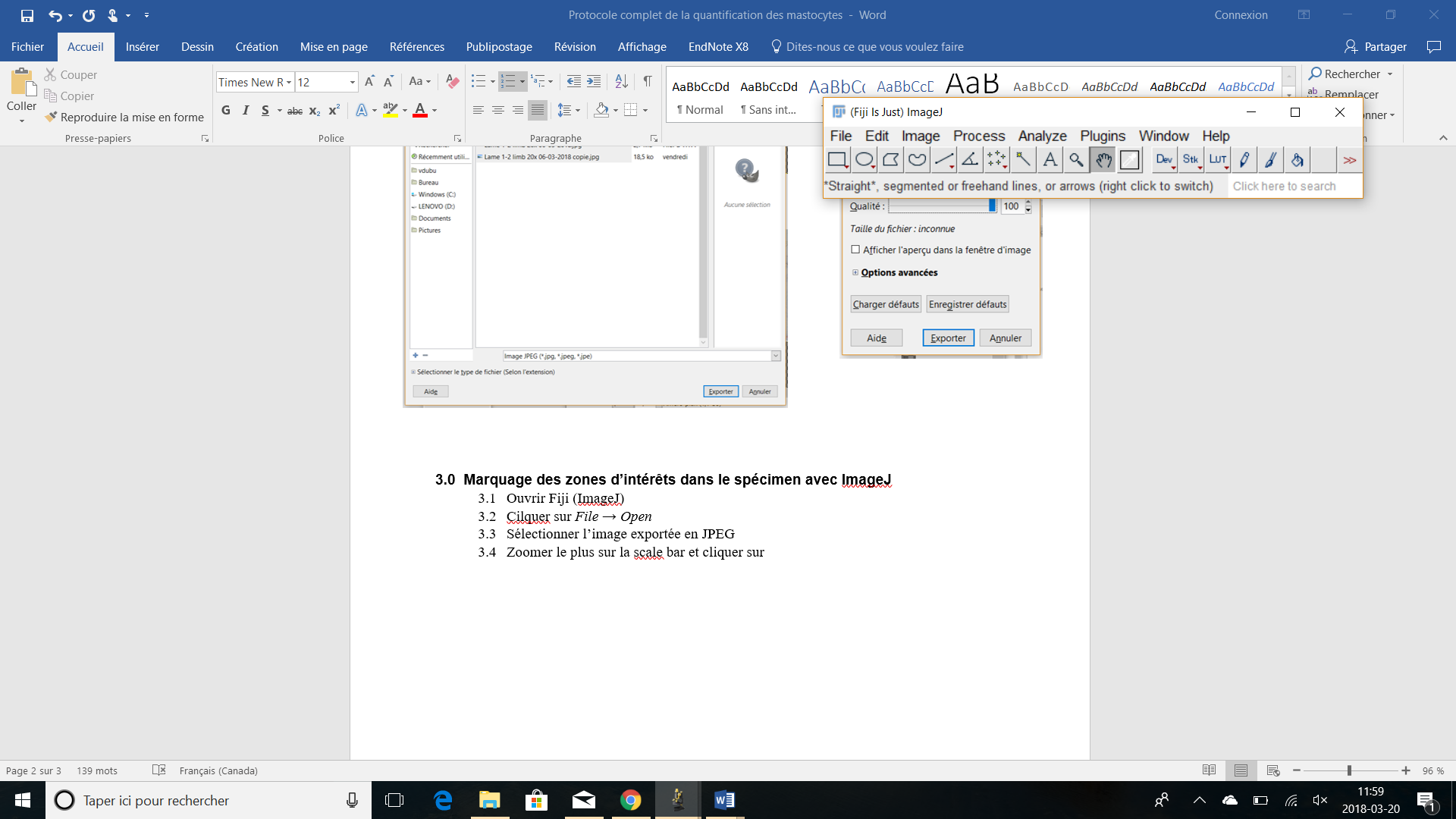
Open an HPF
  2. Open the corresponding scale bar, then select the tool
  3. Trace a line over the scale bar, then click on **Analyze → Set a scale**, enter the corresponding length in µm, check **Global**, then click on **OK**

*
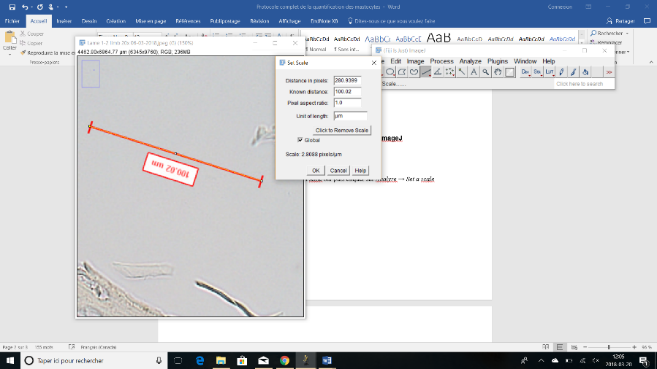
*

- 1.
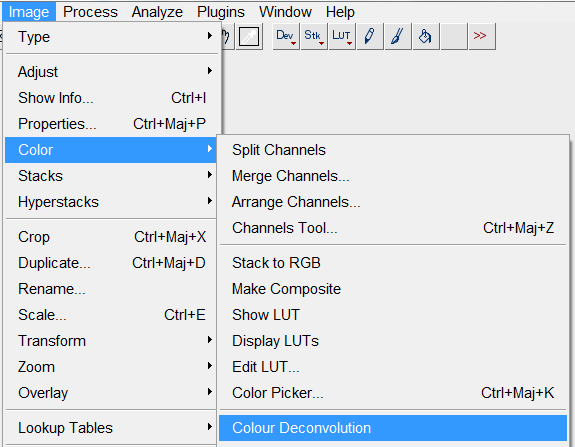
Dissociate the HPF in 3 colours by clicking on **Image → Color → Colour Deconvolution**
  2. Select **H DAB → OK**


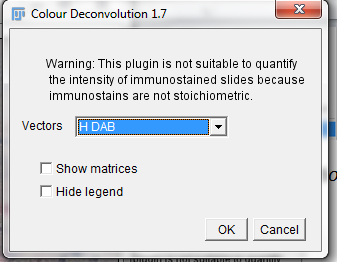


Obtaining 3 images: Color 1 = H&E, Color 2 = DAB


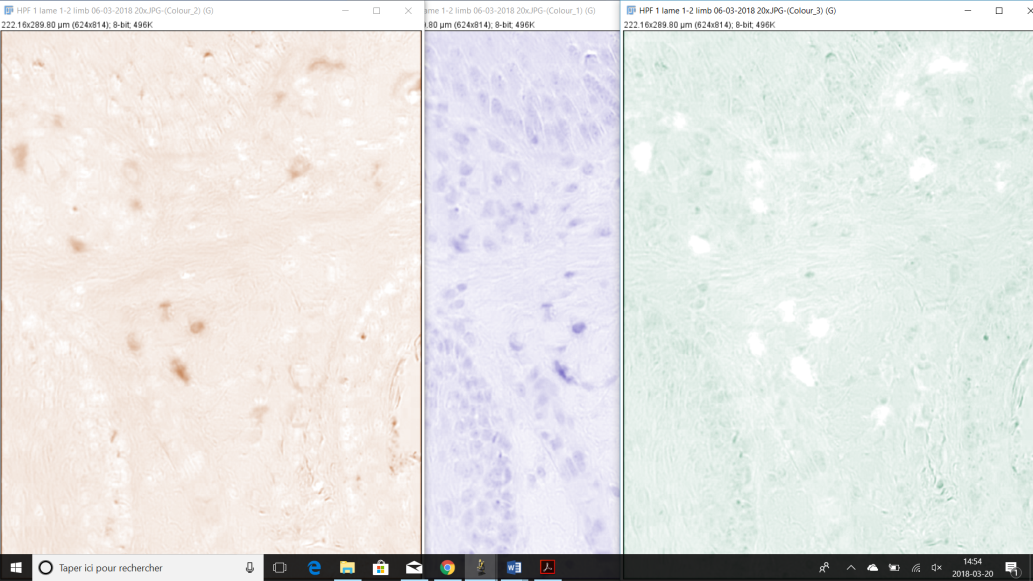


- 1.
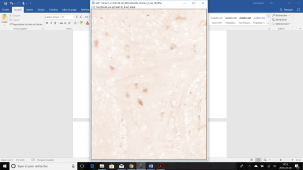
Choose the DAB image →
  2. o in **Process → Binary** the click on **Make binary**


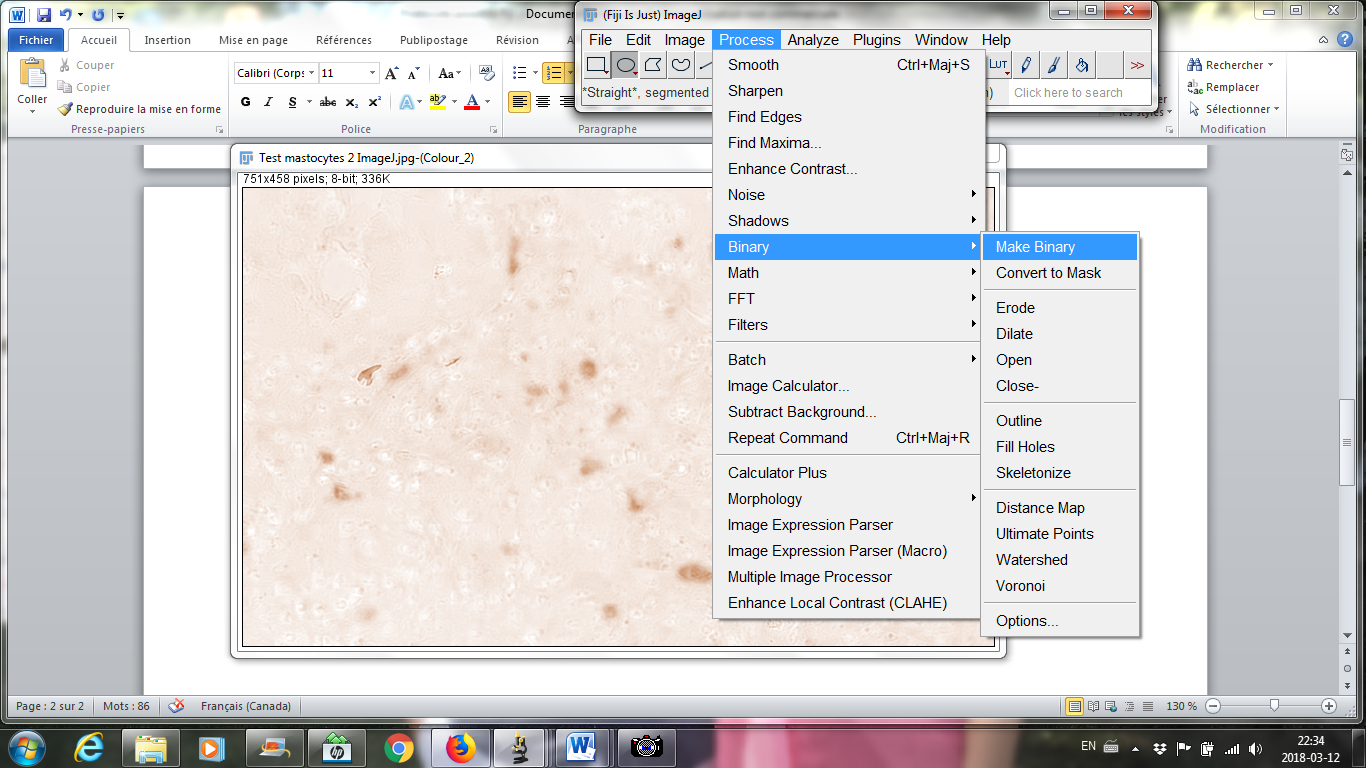


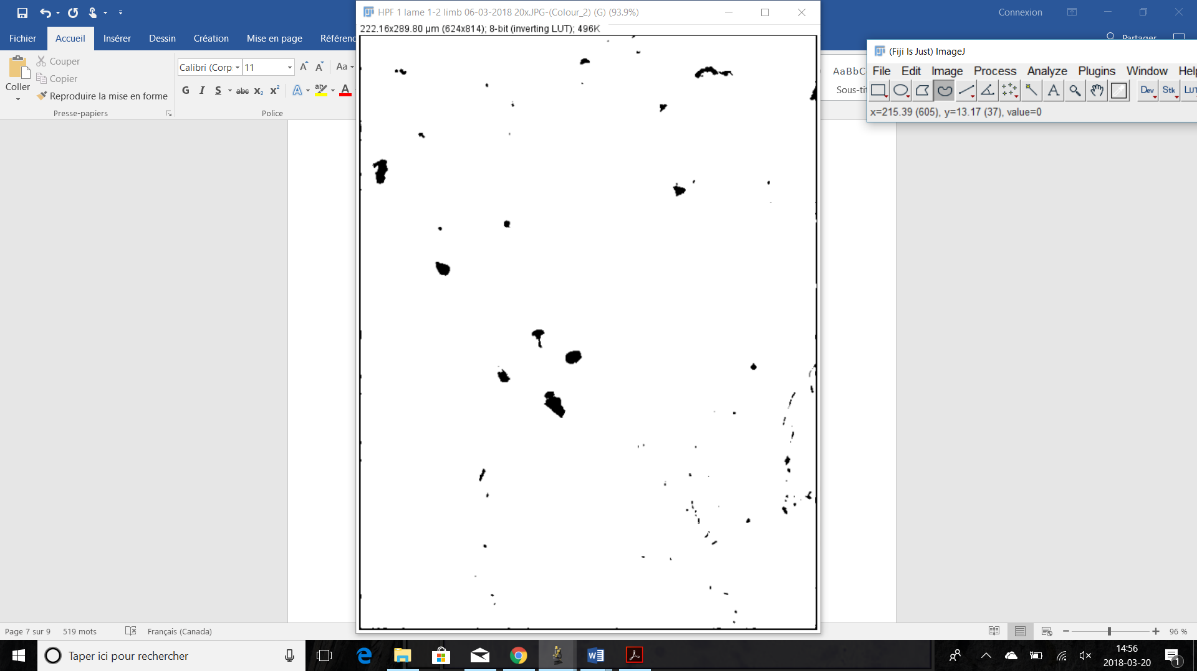
Result →

- 1. If epidermis, blood vessels, sweat/sebaceous glands and/or hair follicles are present, select
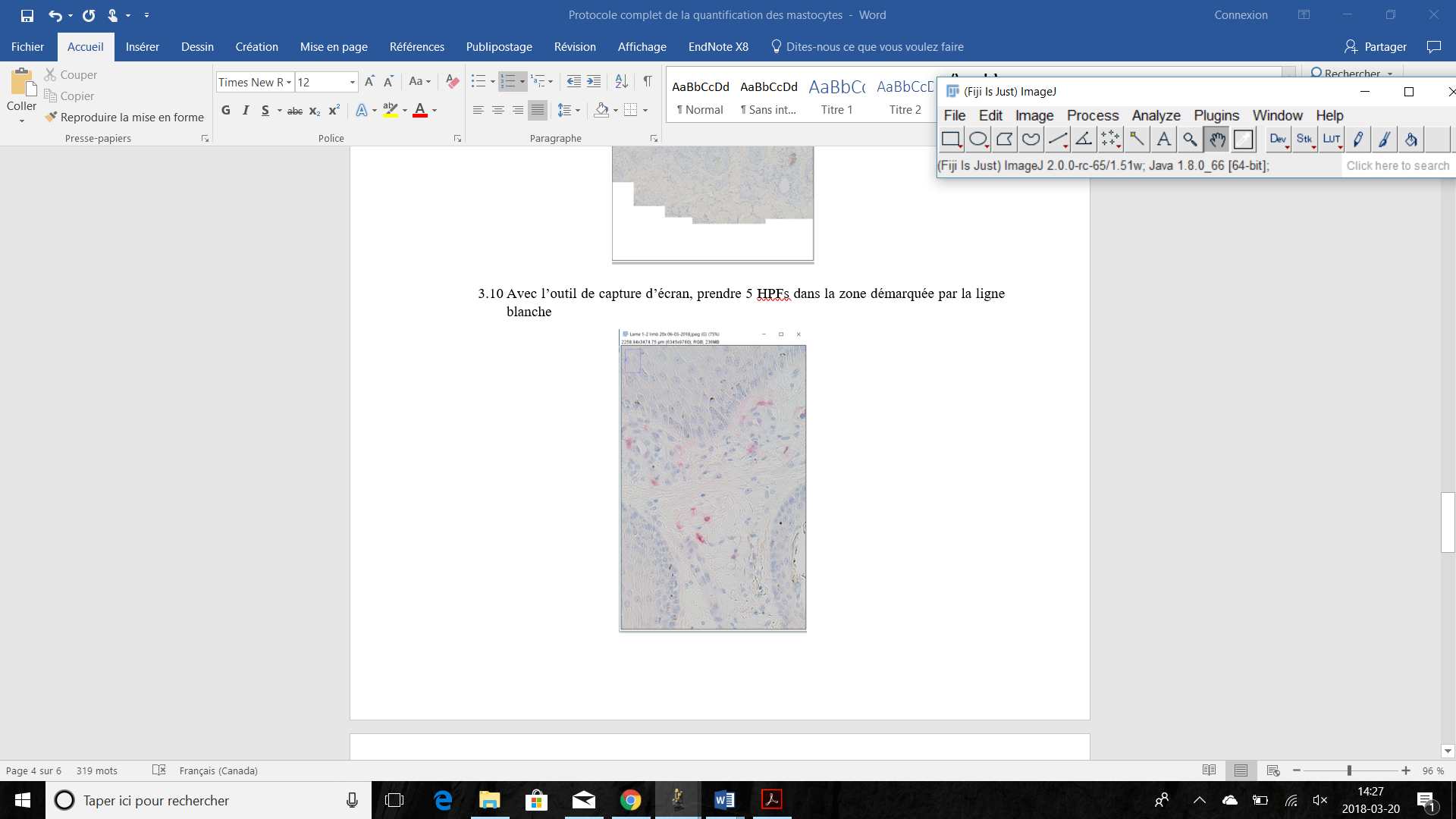
 and draw around undesirable zones to delete, then click on **Erode** until zones are completely disappeareRemove selection and click on **Erode** until background had completely disappeared (only stained cells are remaining)
  2.
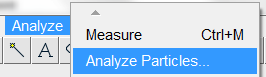
Go to **Analyze** and click on **Analyze particles**
  3. Write the minimal and maximal cell area to consider (**Between 8 et 300 µm²**), go to **Show***,* select **Overlay Masks** and check **Display results**, **Summarize** and **Record stats** → click on *OK*


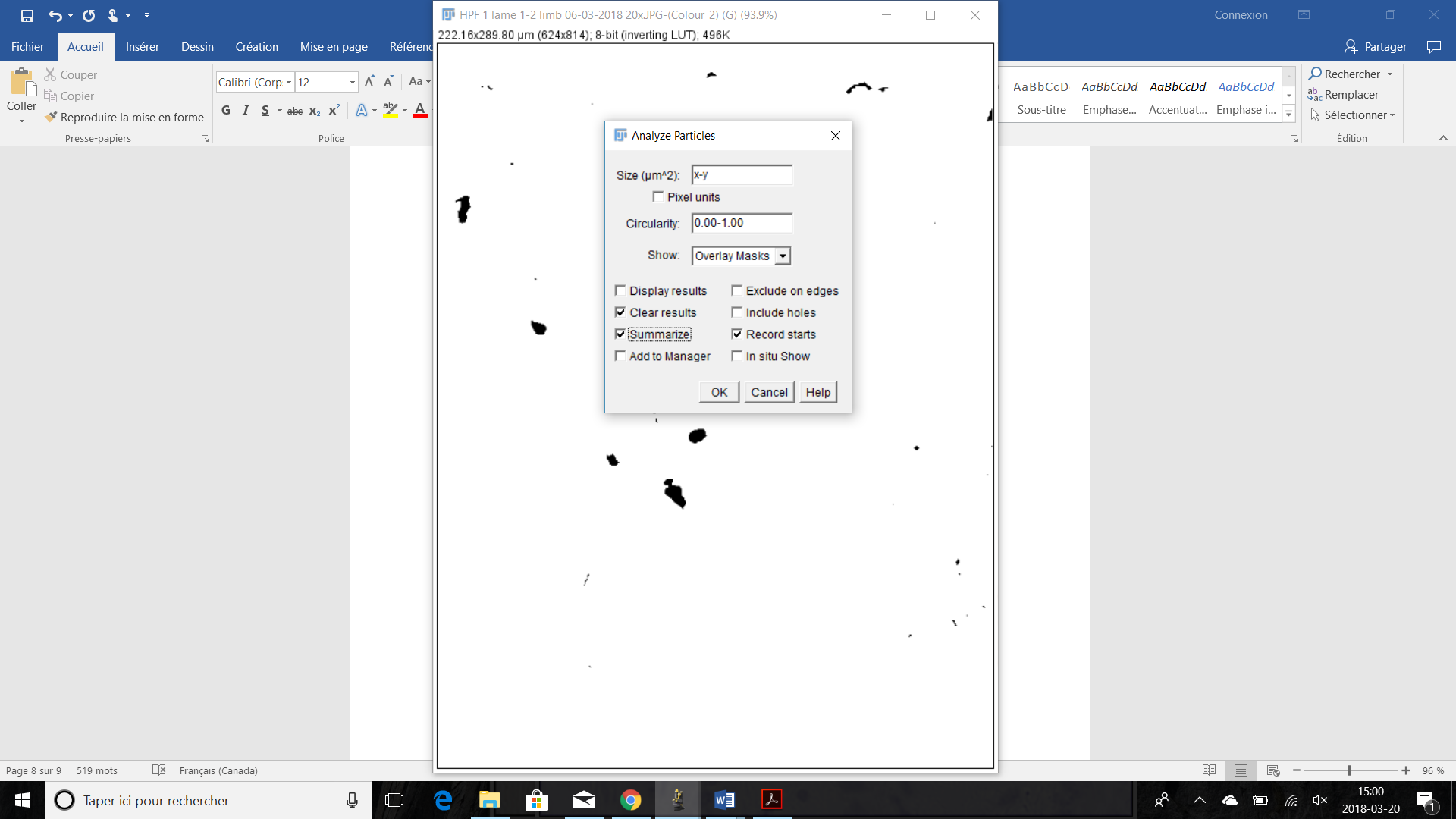


- 1. Result → number of counted cells, % staine surface and others


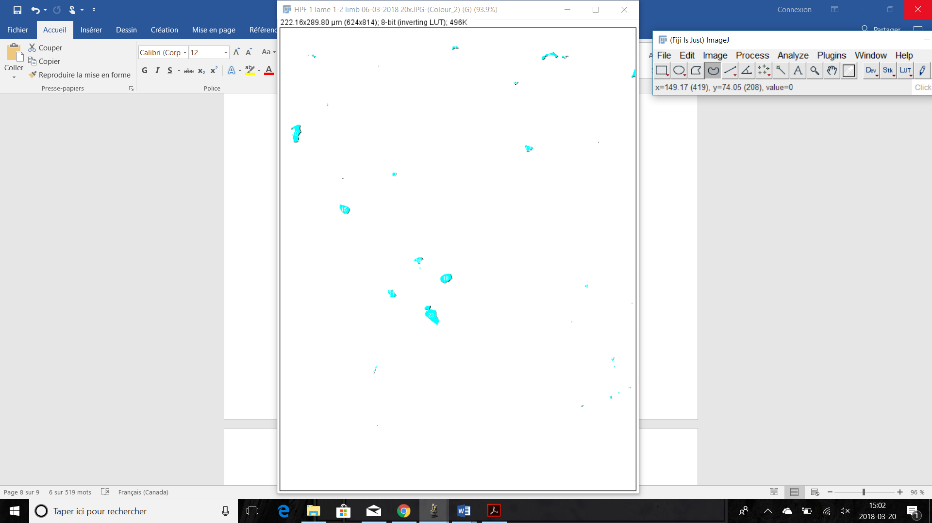


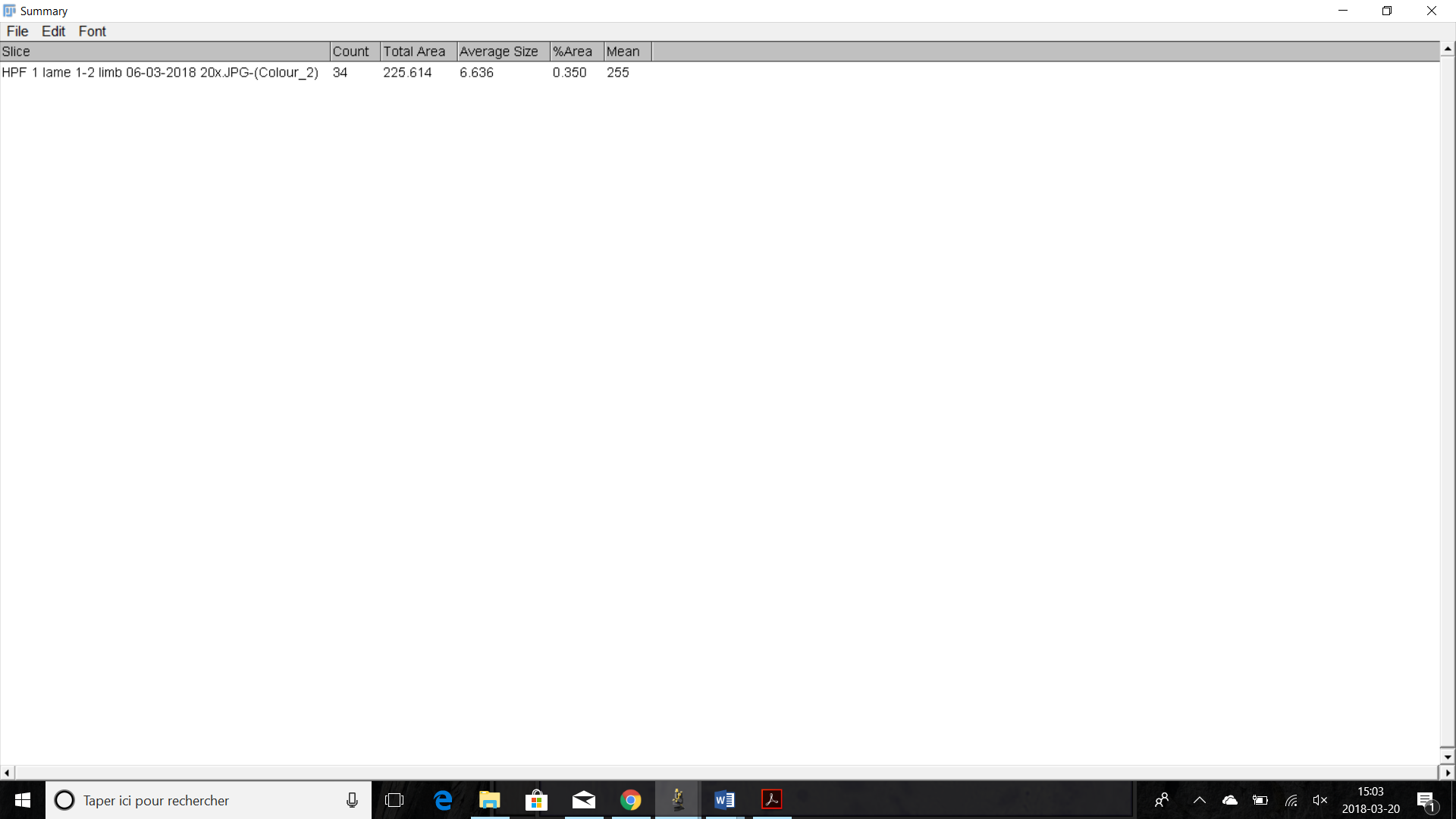


1. **Mast cell expression in number of mast cells/mm²:**
   1. Select
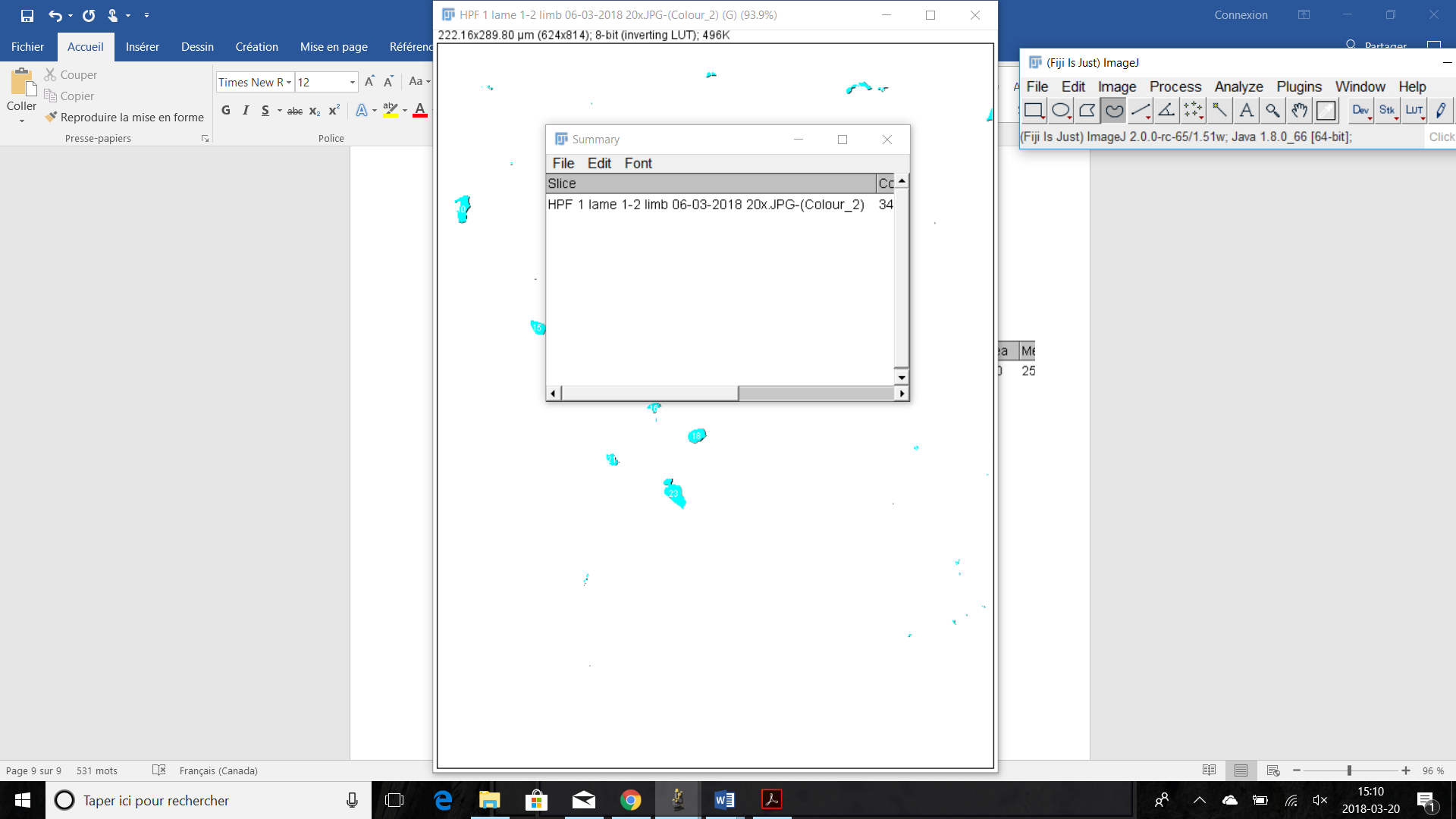
 or
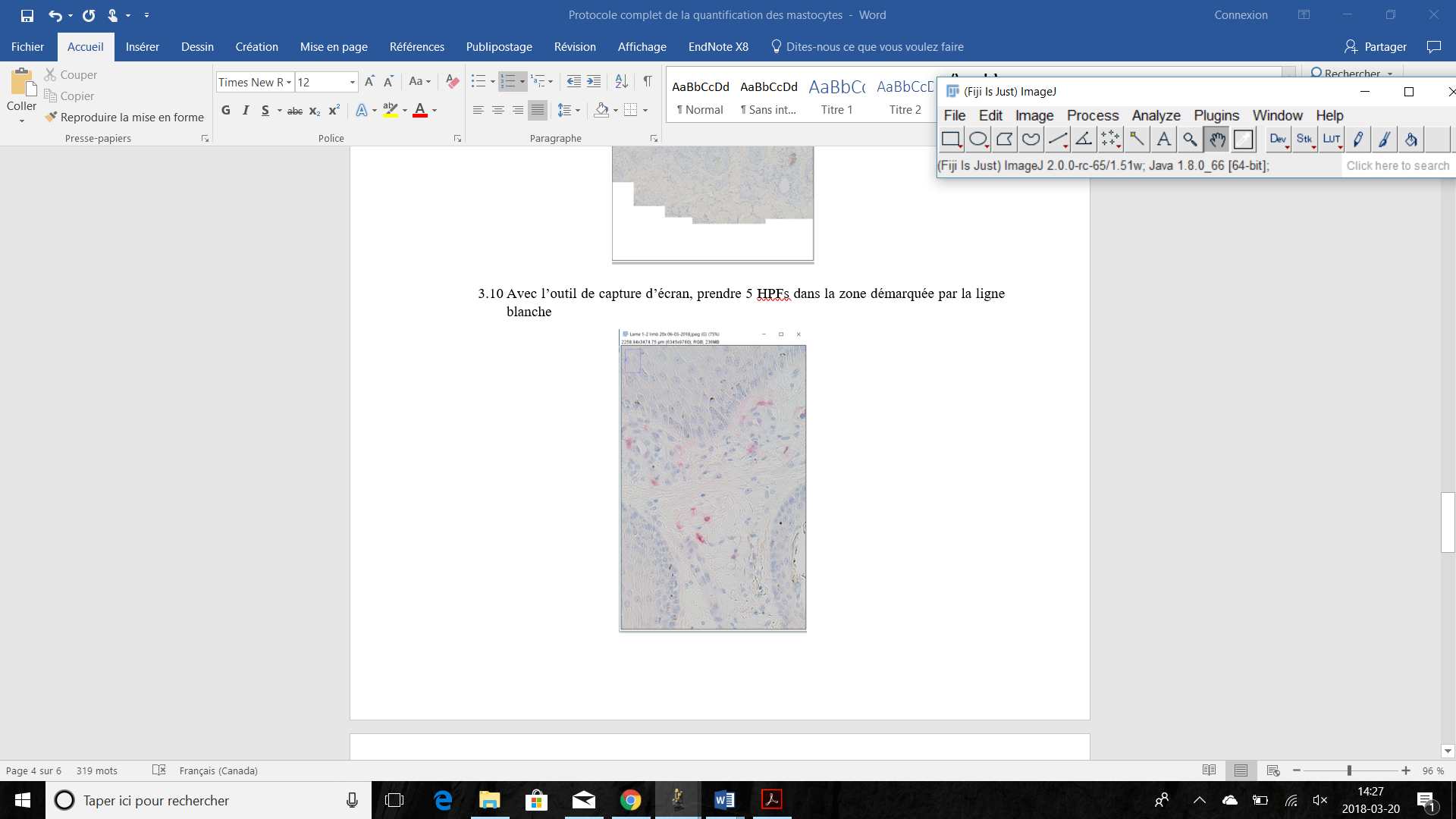
 then draw around HPF borders that just has been quantified, then push on **Ctrl + m** to measure the HPF area


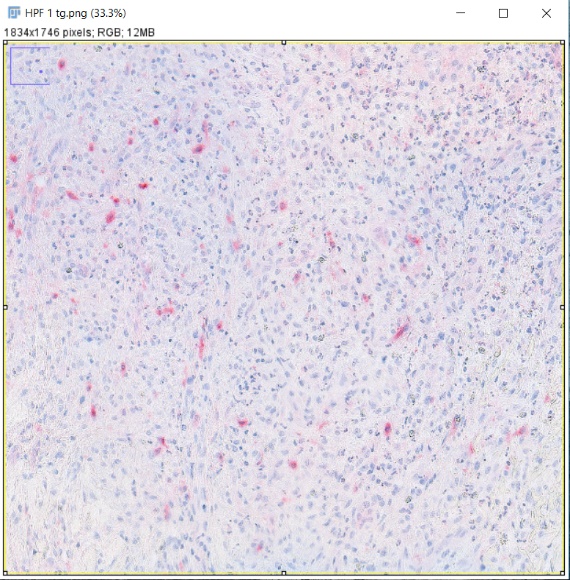

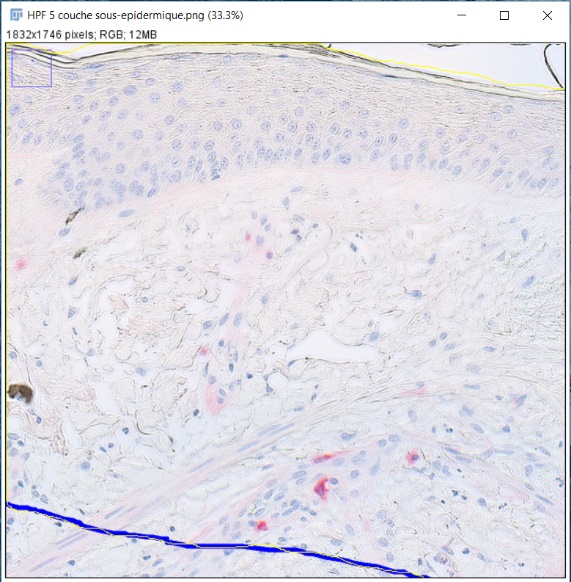


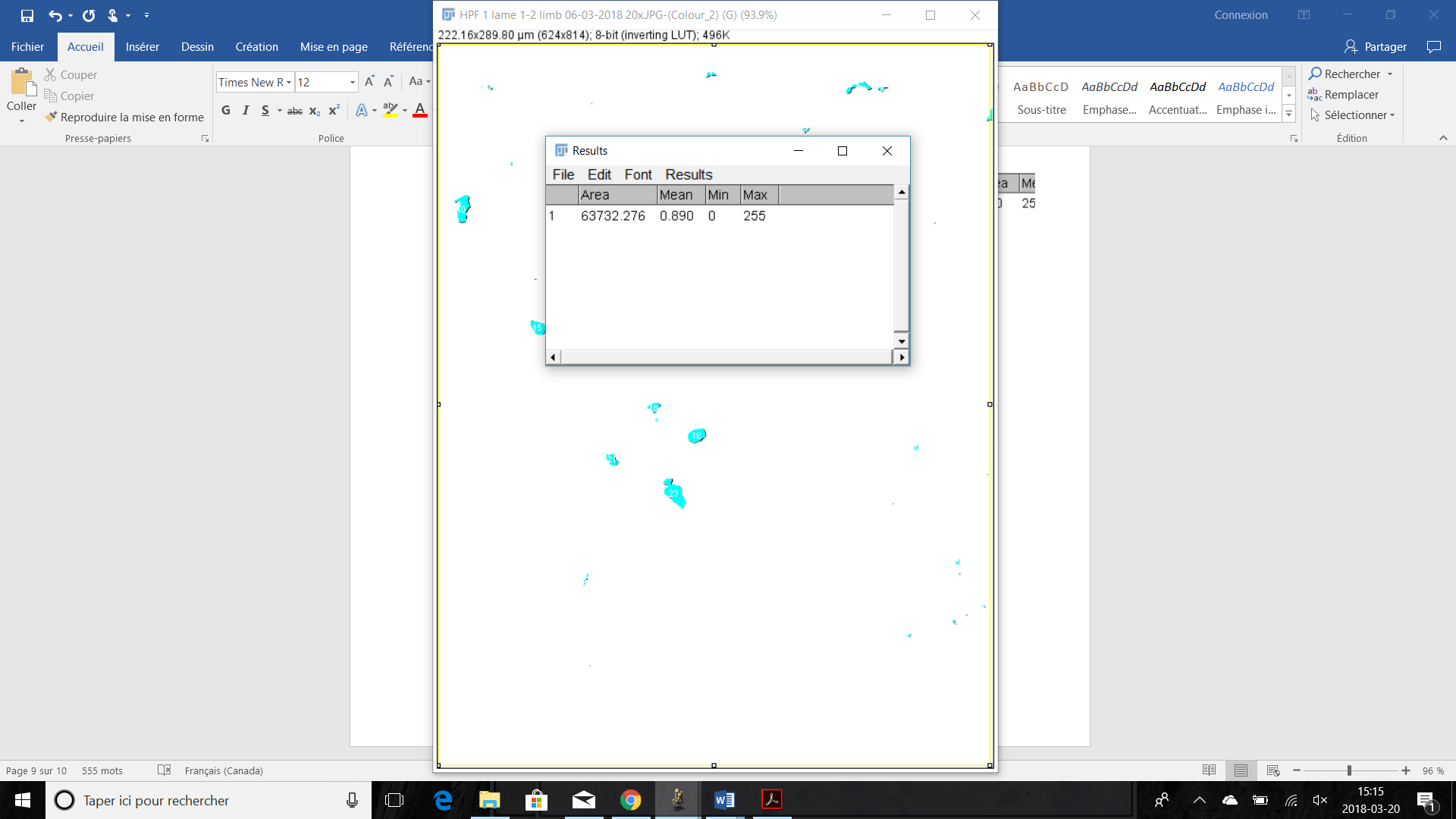


- 1. Transform the area measured in mm² (divide the area by 1 000^2^ = 1 000 000)
  2. Divide the mast cell number by the area calculated in mm²
