# Supplementary material for: Development of a computer-based quantification method for immunohistochemically-stained tissues and its application to study mast cells in equine wound healing (proof of concept)
Source: BMC Vet Res. 2020 Jul 2;16:228. doi: 10.1186/s12917-020-02444-x (PMC7330934; doi:10.1186/s12917-020-02444-x)
Supplement: Supplementary file 3 — Additional file 3. Establishment of protocol of mast cell area interval. This document illustrates how the range of mast cell area interval was established by using the ImageJ software program for the later quantification. [file 12917_2020_2444_MOESM3_ESM.docx]

**Supplementary information 3**

***Establishment protocol of mast cell area interval***

1. Select 6 HPFs taken from different horses and skin layers (2 subepidermal layer, 2 deep dermal layer et 2 from granulation tissue).
2. Open Fiji and click on **File → Open** and open an HPF with its scale bar.
3. Select the scale bar image and zoom as much as possible.
4.
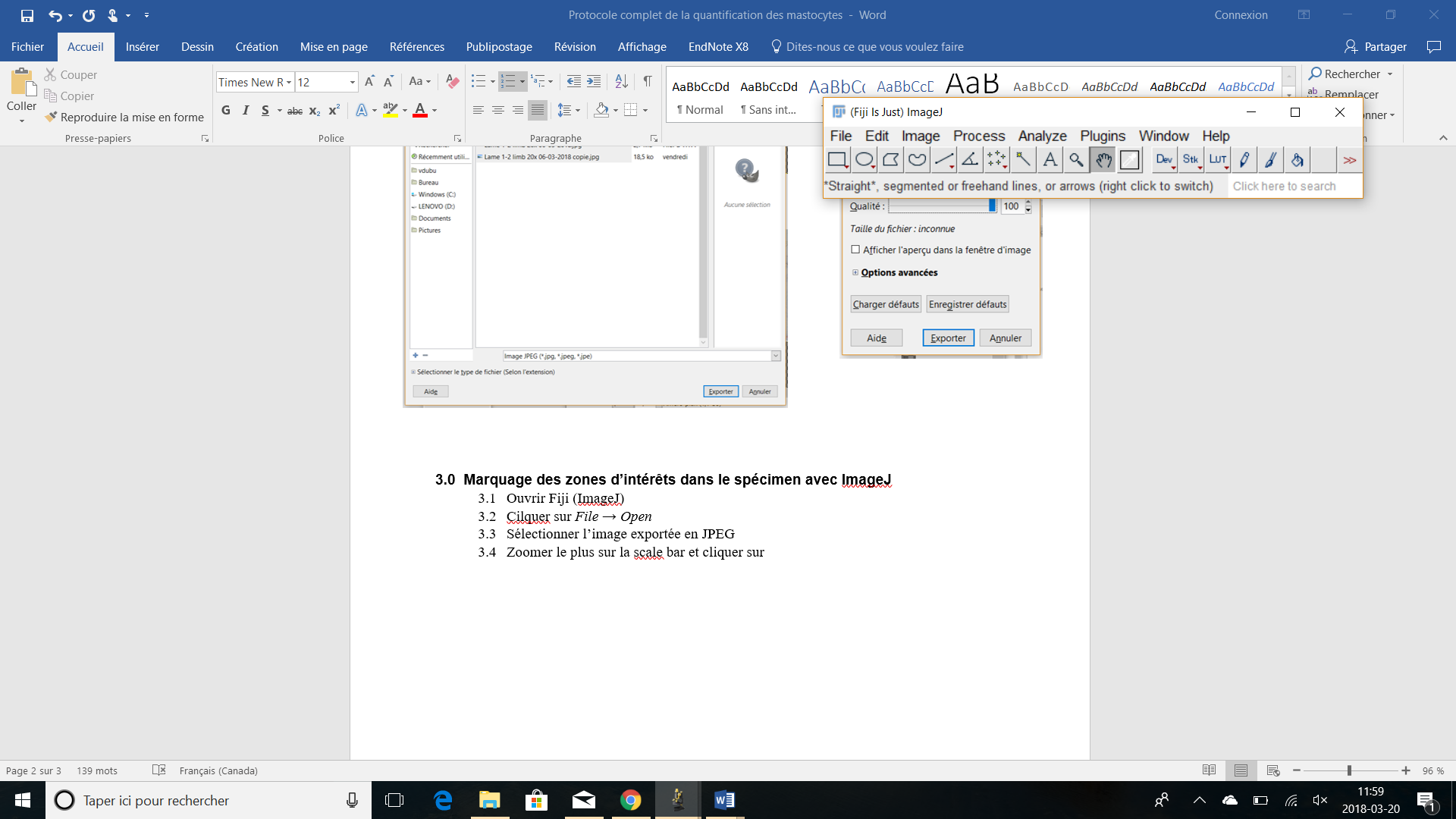
Click on and trace a line over the scale bar.
5. Then, click on **Analyze → Set a scale** and check on **Global** to make sure the measurement will be applied on the next images.
6. Choose the biggest mast cell on the image, then select the tool
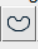
 .
7. Trace mast cell circumference and push on **Ctrl + m** to measure its area.
8. Repeat steps 6 and 7 for the smallest mast cell on the image, then for an average mast cell.
9. Write data in an Excel document.
10. Repeat steps 2 to 9 for the other 5 HPFs.
11. Test the method on few images that have not been used to establish mast cell areas.
12. Establish the range of area to consider.
