# Supplementary material for: Development of a computer-based quantification method for immunohistochemically-stained tissues and its application to study mast cells in equine wound healing (proof of concept)
Source: BMC Vet Res. 2020 Jul 2;16:228. doi: 10.1186/s12917-020-02444-x (PMC7330934; doi:10.1186/s12917-020-02444-x)
Supplement: Supplementary file 4 — Additional file 4 Table S1. Numbers and characteristics of the samples included in the study. Specimen characteristics are grouped in a table to show which specimens were included or excluded in the study and why. [file 12917_2020_2444_MOESM4_ESM.docx]

**Table S1: Numbers and characteristics of the samples included in the study**

| Horse ID | Sex | Age (years) | Day of sampling post wounding | Number of specimens included | |
| --- | --- | --- | --- | --- | --- |
|  |  |  |  | Thoracic wounds | Limb wounds |
| Horse 1 | Female | 15 | 1 | 1 | 1 |
|  |  |  | 3 | 1 | 1 |
|  |  |  | 8 | 1 | * |
|  |  |  | 17 | 1 | * |
| Horse 2 | Female | 10 | 1 | 1 | 1 |
|  |  |  | 3 | 1 | NT |
|  |  |  | 8 | 1 | NT |
|  |  |  | 17 | 1 | NT |
| Horse 3 | Female | 5 | 1 | 1 | 1 |
|  |  |  | 3 | 1 | 1 |
|  |  |  | 8 | 1 | 1 |
|  |  |  | 17 | 1 | N/A |
| Horse 4 | Female | 5 | 1 | 1 | 1 |
|  |  |  | 3 | 1 | 1 |
|  |  |  | 8 | 1 | 1 |
|  |  |  | 17 | 1 | 1 |
| Total |  |  |  | 16 | 10 |

*, specimen harvested but EGT-affected; NT, specimen excluded because it received a negative pressure wound therapy treatment, N/A, non-applicable. Horses did not come with a known history, so age was estimated from the dentition.
